# Fracture outcomes following intravenous pamidronate initiation in children with osteogenesis imperfecta: a multicenter retrospective cohort study from Bucharest, Romania

**DOI:** 10.3389/fped.2026.1973795

**Published:** 2026-10-21

**Authors:** Alexandru Dinulescu, Mara-Elena Stăiculescu, Irina Dijmărescu, Mirela-Luminița Pavelescu, Daniela Păcurar, Alexandru Ulici

**Affiliations:** 1 Department of Pediatrics, “Carol Davila” University of Medicine and Pharmacy, Bucharest, Romania; 2 Department of Pediatrics, Emergency Hospital for Children “Grigore Alexandrescu”, Bucharest, Romania; 3 Department of Pediatric Orthopedics, “Carol Davila” University of Medicine and Pharmacy, Bucharest, Romania; 4 Department of Pediatric Orthopedics, Emergency Hospital for Children “Grigore Alexandrescu”, Bucharest, Romania

**Keywords:** bisphosphonate, fracture rate, osteogenesis imperfecta, pamidronate, pediatric

## Abstract

**Introduction:**

Osteogenesis imperfecta (OI) is a rare inherited disorder characterized by skeletal fragility and recurrent fractures. Intravenous pamidronate is widely used in pediatric OI, but long-term real-world data from Eastern Europe remain limited. We compared fracture incidence before and after pamidronate initiation in Romanian children with OI.

**Methods:**

We conducted a 20-year retrospective multicenter before-and-after cohort study in three tertiary pediatric centers in Bucharest, Romania. Fracture counts and person-years before and after treatment initiation were analyzed using negative binomial generalized estimating equations (GEE), accounting for repeated observations within patients and including log person-years as an offset. The model included treatment period, Sillence type, sex, and age at pamidronate initiation.

**Results:**

Forty-nine children with OI receiving intravenous pamidronate were included. The cohort included 28 females (57.1%) and 21 males (42.9%). Median age at diagnosis was 24.5 months (IQR, 0.25–81.25), and pamidronate was initiated at a median age of 45 months (IQR, 15.5–95). Before initiation, 198 fractures occurred over 250.3 person-years, compared with 208 fractures over 359.3 person-years afterward, corresponding to incidence rates of 0.79 and 0.58 fractures per person-year, respectively. In the adjusted GEE model, fracture incidence was lower during the post-pamidronate period (IRR 0.49, 95% CI 0.35–0.68; *p* < 0.001). Compared with type I OI, fracture incidence was higher in type III (IRR 2.09, 95% CI 1.49–2.92; *p* < 0.001) and type IV (IRR 1.72, 95% CI 1.01–2.94; *p* = 0.048). Sex was not associated with fracture incidence. Older age at pamidronate initiation was associated with lower fracture incidence (IRR 0.992 per month, 95% CI 0.989–0.996; *p* < 0.001). Adjustment for corrective osteotomies with telescopic intramedullary rodding procedures did not materially change the findings.

**Conclusions:**

Fracture incidence was substantially lower after pamidronate initiation, including after accounting for repeated within-patient observations and clinical characteristics. Although the observational design prevents causal attribution to pamidronate, these findings provide long-term real-world data from an Eastern European pediatric OI cohort and support prospective studies incorporating age-dependent fracture risk and time-varying exposures.

## 1 Introduction

Osteogenesis imperfecta (OI) is a rare inherited connective tissue disorder that is characterized by diminished bone strength and increased skeletal fragility, resulting in recurrent fractures, bone deformities, growth impairment, and functional disability (1, 2). The incidence of OI is estimated between 1 in 15 000 to 1 in 20 000 live births worldwide (3, 4). Most cases are caused by pathogenic variants affecting type I collagen synthesis, predominantly involving the *COL1A1* and *COL1A2* genes, although several non-collagen-related genetic forms have also been described (5–8).

Clinical manifestations vary significantly, ranging from mild forms with few fractures and minimal deformities to severe progressive forms associated with significant skeletal deformities, numerous fractures and impaired ambulation (9–11). The traditional Sillence classification remains widely used in clinical practice and categorizes OI into several major phenotypic subtypes according to disease severity, fracture burden, skeletal deformities and extra-skeletal manifestations (12, 13). In children, recurrent long-bone fractures, progressive lower limb deformities, scoliosis, growth impairment, dentinogenesis imperfecta, blue sclerae, and hearing impairment represent common clinical features (14, 15).

Management of OI has evolved into a multidisciplinary approach combining medical treatment, orthopedic surgery, physiotherapy, and rehabilitation. Among pharmacological therapies, intravenous pamidronate has been the most extensively used bisphosphonate in children for more than two decades (16–18). The first landmark pediatric study evaluating cyclical intravenous pamidronate therapy in severe OI was published by Glorieux et al. in 1998 and demonstrated significant improvements in bone mineral density, vertebral morphology, mobility, and fracture incidence in affected children (19). Pamidronate acts by inhibiting osteoclast-mediated bone resorption, leading to increased bone mass and improved skeletal stability (18). There are multiple studies that reports its beneficial effect in lumbar spine bone mineral density, fracture reduction and long-term skeletal outcomes (20–24). Systematic reviews have consistently shown improvements in bone mineral density, while evidence for fracture reduction has been less conclusive because most randomized studies were not adequately powered for fracture-related outcomes (17, 25). Even though intravenous pamidronate has historically been widely used in children with OI, other bisphosphonate therapy, particularly zoledronic acid, are increasingly used in pediatric practice, and treatment protocols vary between countries and specialized centers (26–28). Our present study will specifically evaluate patients treated with intravenous pamidronate, reflecting the treatment practice of the participating Romanian centers during the study period. In addition to pharmacological therapy, multidisciplinary management including orthopedic surgery, intramedullary rodding, physiotherapy, and rehabilitation plays a major role in improving mobility and long-term functional outcomes (29–31).

Although intravenous pamidronate has been used in pediatric OI for more than two decades, evidence from routine clinical practice remains relatively limited, particularly in Eastern European countries. Unlike controlled clinical trials, real-world cohorts reflect the heterogeneity of patients encountered in everyday practice, including variations in disease severity, timing of treatment initiation, access to orthopedic surgery, physiotherapy, genetic testing, and long-term follow-up. Understanding treatment outcomes under these conditions is essential for optimizing multidisciplinary care and identifying the clinical characteristics associated with greater therapeutic benefit.

The primary objective of this multicenter cohort study was to evaluate changes in fracture incidence rate following initiation of intravenous pamidronate therapy in children with OI treated at three tertiary referral centers in Romania. Secondary objectives included exploring clinical factors associated with fracture incidence, including OI subtype, sex, age at pamidronate initiation, and history of corrective osteotomies with telescopic intramedullary rodding procedures.

## 2 Materials and methods

### 2.1 Study design and setting

This retrospective multicenter before-and-after cohort study was conducted over a 20-year period (January 1, 2001–December 31, 2021) in 3 tertiary pediatric referral centers: “Grigore Alexandrescu” Emergency Clinical Hospital for Children, “Marie Curie” Emer-gency Clinical Hospital for Children and “Victor Gomoiu” Clinical Hospital for Children, from Bucharest, Romania. These institutions are major tertiary referral centers for orthopedic diseases and among the largest pediatric hospitals in the country, providing care for children with rare and complex conditions, including osteogenesis imperfecta. The study compared fracture burden before and after initiation of intravenous pamidronate therapy with each patient contributing observations to both the pre- and post-pamidronate initiation periods, thereby reducing between-patient variability related to disease severity.

Intravenous pamidronate was used off-label for pediatric OI. Because the cohort included patients treated from the early 2000s onward, the therapeutic framework evolved during the study period. Earlier treatments were initiated as part of specialist clinical care based on the available evidence and pediatric OI treatment protocols at that time, while bisphosphonate therapy for pediatric OI was subsequently incorporated into the Romanian national therapeutic protocol, which provided standardized eligibility, dosing, and monitoring recommendations (19, 20, 32). The decision to initiate intravenous pamidronate was individualized according to the clinical course, fracture burden, severity of skeletal involvement, hospital treatment availability and the clinical judgment of the treating physician. Given the long study period, treatment eligibility was not based on a single uniform numerical threshold throughout the entire cohort. In particular, a predefined number of long-bone fractures, a specific vertebral fracture criterion, or a fixed DXA threshold was not consistently used or documented. Treatment practices became more standardized in later years following the introduction of the Romanian national therapeutic protocol for OI (32).

### 2.2 Study population

We included children (aged <18 years) diagnosed with osteogenesis imperfecta during the study period. Diagnosis was established based on clinical and radiological findings, with genetic confirmation where available. In patients without molecular confirmation, the diagnosis was established by the treating physician based on the characteristic clinical phenotype, fracture history, and radiological findings. Alternative causes of bone fragility and impaired mineralization were evaluated as clinically appropriate using biochemical and radiological investigations available at the time. These included relevant differential diagnoses such as hypophosphatasia and hypophosphatemic rickets (33). Because of the retrospective design and the long study period, the diagnostic work-up was not fully standardized across all patients and participating centers. Molecular confirmation was not required for study inclusion. In two patients, targeted testing of COL1A1 and COL1A2 did not identify a pathogenic variant; broader multigene panel testing or whole-exome sequencing was not subsequently performed. These patients were retained in the cohort because the diagnosis of OI had been established clinically and radiologically by the treating physicians.

Patients were included if they:

- received intravenous bisphosphonate therapy
- had documented fracture history before treatment initiation
- had available follow-up data after treatment initiation
- had at least one year between birth and pamidronate initiation, with sufficient documented fracture history to estimate the pre-initiation fracture incidence

Patients were excluded if they had:

- incomplete medical records;
- uncertain diagnosis of OI;
- absence of documented fractures before treatment initiation;
- no exposure to intravenous pamidronate;
- insufficient follow-up to calculate post- pamidronate initiation period fracture rates.

We excluded 37 patients, remaining 49 patients that were included in this study (Figure 1). The reasons for exclusion were predefined before data extraction.

**Figure 1:**
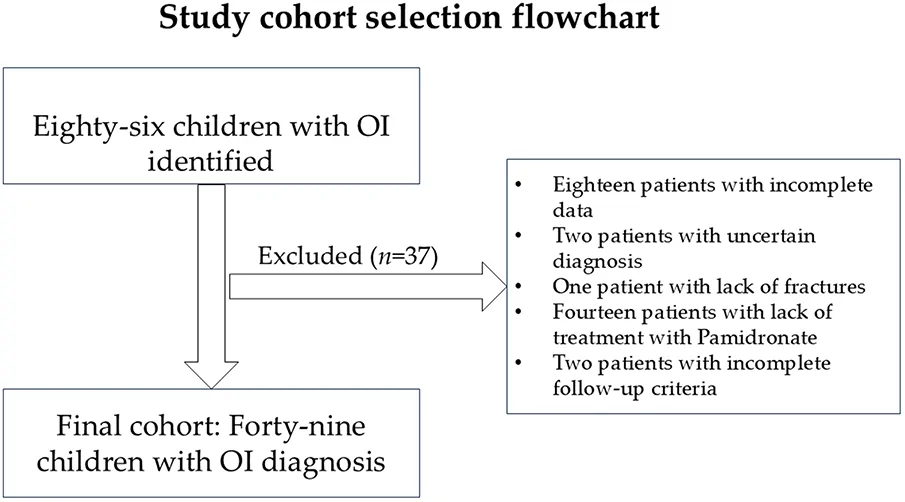
Flowchart of patient identification, eligibility assessment, and selection of the final cohort of children with osteogenesis imperfecta receiving intravenous pamidronate.

### 2.3 Data collection

Data were retrospectively extracted from electronic and paper medical records using a standardized data collection form. The following variables were collected: sex, age at diagnosis, Sillence OI subtype, age at initiation of pamidronate therapy, duration of pamidronate treatment, duration of follow-up, fracture history, and orthopedic and rehabilitation interventions.

The number of documented fractures and the corresponding observation time were recorded separately for the periods before and after initiation of pamidronate therapy. The occurrence of corrective osteotomies with telescopic intramedullary rodding procedures was also recorded.

### 2.4 Definitions

For each patient, observation time was calculated separately for the pre-pamidronate and post-pamidronate periods. The pre-pamidronate period extended from birth until initiation of pamidronate therapy, while the post-pamidronate initiation period extended from treatment initiation until the end of available follow-up: for patients who were younger than 18 years at the time of data collection, the last available follow-up was the most recent clinical assessment before database closure (the database was closed on 31 May 2026) and for patients who reached adulthood (over 18 years-old) during the study period, it was their last pediatric assessment before transfer to adult healthcare services. Patients who had not yet reached 18 years at the time of data collection remained under pamidronate treatment and pediatric follow-up. Patients lost to follow-up, for whom longitudinal treatment and fracture data could not be reliably reconstructed, were excluded. Fracture incidence was calculated by dividing the number of documented fractures occurring during each observation period by the corresponding person-time at risk. Person-time was expressed in years.

Intravenous pamidronate was administered in cyclic regimens consisting of 0.5–1 mg/kg/day for three consecutive days, repeated every 3–4 months (19, 34).

Orthopedic surgery referred to corrective osteotomies with telescopic intramedullary rodding. In the present cohort, all recorded corrective osteotomies with telescopic intramedullary rodding procedures were performed after initiation of pamidronate therapy. Therefore, surgical interventions were considered potential contributors to fracture outcomes observed during the post-initiation follow-up period and were interpreted as part of the multidisciplinary management of OI rather than as independent treatment effects. All documented intramedullary rodding procedures in the included cohort involved the lower extremities; no upper-extremity rodding procedures were recorded.

### 2.5 Statistical analysis

Statistical analyses were performed using IBM SPSS Statistics version 25 (IBM Corp., Armonk, NY, USA). Continuous variables were assessed for normality using the Shapiro–Wilk test and are presented as mean ± standard deviation (SD) or median and interquartile range (IQR), as appropriate. Categorical variables are presented as frequencies and percentages.

Crude fracture incidence rates before and after pamidronate initiation were calculated as the total number of fractures divided by the corresponding person-years of observation. The pre-pamidronate observation period extended from birth to initiation of pamidronate therapy, whereas the post-pamidronate initiation period extended from treatment initiation to the end of the available follow-up. The logarithm of person-years was used as an offset in count regression analyses to account for differences in observation time between periods and among patients.

Because each patient contributed two observations (one pre-pamidronate and one post-pamidronate) and fracture counts exhibited overdispersion, the primary multivariable analysis was performed using a generalized estimating equations (GEE) negative binomial regression model with a log link function. Patient identifier was specified as the subject variable and treatment period (PRE vs. POST) as the within-subject variable, thereby accounting for the correlation between repeated observations from the same pa-tient. An exchangeable working correlation structure was specified.

The primary GEE model included treatment period, Sillence OI subtype, sex, and age at pamidronate initiation. Sillence type I, male sex, and the pre-pamidronate period were used as reference categories. Because the pre- and post-pamidronate initiation periods correspond to different stages of childhood and age-related changes in fracture risk represent a potential source of confounding, age at pamidronate initiation was included as a patient-level covariate in the multivariable model (as a continuous variable in months). However, this variable represents age at the start of treatment and does not account for the time-varying effect of chronological age, growth, or skeletal maturation during follow-up. The GEE model accounts for the correlation between repeated pre- and post-treatment observations within the same patient, but it does not eliminate potential age-related confounding inherent to the before-and-after design. The period effect was interpreted as an association with fracture incidence rather than as an isolated causal effect of pamidronate.

Effect estimates are presented as incidence rate ratios (IRRs), obtained by exponentiating the regression coefficients, together with 95% confidence intervals (CIs). An IRR be-low 1 indicates a lower fracture incidence rate, whereas an IRR above 1 indicates a higher fracture incidence rate relative to the reference category.

As a sensitivity analysis, the GEE model was additionally adjusted for whether patients underwent a corrective osteotomies with telescopic intramedullary rodding procedures procedure. Because the precise timing of orthopedic procedures relative to individual fractures could not be reliably reconstructed from the retrospective records, Corrective osteotomies with telescopic intramedullary rodding procedures surgery could not be modeled as a time-varying exposure; therefore, this analysis was considered exploratory. Physiotherapy was not included in the multivariable model because its timing and intensity relative to the pre- and post-pamidronate initiation periods could not be reliably established.

All statistical tests were two-sided, and a *p*-value < 0.05 was considered statistically significant.

### 2.6 Ethical considerations

The study was conducted in accordance with the Declaration of Helsinki, and approved by the Ethics Committee of “Grigore Alexandrescu” Emergency Hospital for Children, Bucharest, Romania, with approval number 30, on 09 June 2026. Informed consent was obtained from all subjects involved in the study.

## 3 Results

### 3.1 Study population main characteristics

We found 86 patients in the electronic registers of the 3 hospitals. We excluded 37 patients, remaining 49 patients that were included in this study (Figure 1). The reasons for exclusion were predefined before data extraction. The cohort had 28 females (57.1%) and 21 males (42.9%). The median age at diagnosis was 24.5 (0.25–81.25) months.

According to the Sillence classification, type I OI was the most frequent subtype, with 21 patients (42.9%), followed by type III (17, 34.7%), type IV (9, 18.4%), and type V (2, 4.1%). Genetic testing was not performed in 32 patients (65.3%); among those tested, pathogenic variants were identified predominantly in COL1A1 and COL1A2.

Corrective osteotomies with telescopic intramedullary rodding procedures were performed in 29 patients (59.2%), and 36 patients (73.5%) received physiotherapy during the observation period. Detailed baseline characteristics are presented in Table 1.

**Table 1:** Demographic and clinical characteristics of 49 children with osteogenesis imperfecta receiving intravenous pamidronate.

Variable	N (%) or Median (IQR)
Sex	
Female	28 (57.1%)
Male	21 (42.9%)
Age at diagnosis	24.5 (0.25–81.25) months
OI type	
Type I	21 (42.9%)
Type III	17 (34.7%)
Type IV	9 (18.4%)
Type V	2 (4.1%)
Genetic testing	
Not performed	32 (65.3%)
Performed, no pathogenic variant	2 (4.1%)
*COL1A1*	9 (18.4%)
*COL1A2*	5 (10.2%)
*IFITM5*	1 (2.0%)
Corrective osteotomies with telescopic intramedullary rodding procedures	
Yes	29 (59.2%)
No	20 (40.8%)
Physiotherapy	
Yes	36 (73.5%)
No	13 (26.5%)

### 3.2 Pamidronate treatment and observation period

Intravenous pamidronate therapy was initiated at a median age of 45 (15.5–95) months. The median duration of pamidronate treatment was 72 (52.5–113.5) months, while the median overall follow-up period was 144 (111.5–185) months. Treatment and follow-up characteristics are summarized in Table 2.

**Table 2:** Age at pamidronate initiation, treatment duration, and follow-up in children with osteogenesis imperfecta.

Variable	Median (IQR)
Time of follow-up (months)	144 (111.5–185)
Age at initiation of pamidronate therapy (months)	45 (15.5–95)
Duration of treatment (months)	72 (52.5–113.5)

### 3.3 Fracture burden before and after pamidronate initiation

The median annual fracture rate during the pre-treatment observation period was 0.80 (0.37–1.63) fractures per person-year. Across the entire cohort, 198 fractures occurred during 250.3 person-years of observation before pamidronate initiation, corresponding to a fracture incidence rate of 0.79 fractures per person-year. Following pamidronate initiation, 208 fractures were recorded over 359.3 person-years, corresponding to an incidence rate of 0.58 fractures per person-year. Thus, the crude fracture incidence rate was significantly lower during the post-initiation period than during the pre-treatment period (IRR 0.73, 95% CI 0.60–0.89; *p* = 0.002), corresponding to an approximately 27% reduction in fracture incidence (Table 3).

**Table 3:** Crude fracture incidence before and after intravenous pamidronate initiation in children with osteogenesis imperfecta.

Variable	Pre-pamidronate initiation	Post-pamidronate initiation
Total fractures	198	208
Person-years	250.3	359.3
Fracture incidence (fractures/person-year)	0.79 (Reference)	0.58 (IRR 0.73, 95% CI 0.60–0.89; *p* = 0.002)

Because each patient contributed one pre- and one post-initiation observation, fracture counts were subsequently analyzed using a negative binomial GEE model to account for within-patient correlation, with person-years included as an offset (Table 4). All 49 patients contributed two observations to the analysis (98 observations in total).

**Table 4:** Factors associated with fracture incidence in children with osteogenesis imperfecta: multivariable negative binomial GEE analysis.

Predictor	IRR	95% CI	*p*-value
Period			
PRE	1.00 (ref.)	—	—
POST	0.49	0.35–0.68	<0.001
Sillence type			
Type I	1.00 (ref.)	—	—
Type III	2.09	1.49–2.92	<0.001
Type IV	1.72	1.01–2.94	0.048
Type V	2.50	0.82–7.59	0.106
Sex			
Male	1.00 (ref.)	—	—
Female	1.23	0.89–1.70	0.213
Age at pamidronate initiation, per month	0.992	0.988–0.996	<0.001

After adjustment for Sillence type, sex, and age at pamidronate initiation, fracture incidence remained significantly lower during the post-pamidronate initiation period than during the pre-pamidronate period (IRR 0.49, 95% CI 0.35–0.68; *p* < 0.001). Thus, the estimated fracture incidence rate during the post-initiation period was approximately 51% lower than that observed during the pre-initiation period, after accounting for the included covariates and the correlation between repeated observations within the same patient.

Compared with Sillence type I, type III was associated with a higher fracture incidence rate (IRR 2.09, 95% CI 1.49–2.92; *p* < 0.001), as was type IV (IRR 1.72, 95% CI 1.01–2.94; *p* = 0.048). Although the point estimate was also higher for type V (IRR 2.50), the confidence interval was wide and the association did not reach statistical significance (95% CI 0.82–7.59; *p* = 0.106), consistent with the very small number of patients with this sub-type.

Sex was not independently associated with fracture incidence (female vs. male: IRR 1.23, 95% CI 0.89–1.70; *p* = 0.213). Older age at pamidronate initiation was associated with a lower overall fracture incidence rate (IRR 0.992 per additional month, 95% CI 0.988–0.996; *p* < 0.001). However, because age at treatment initiation is a patient-level characteristic rather than a time-varying measure of age during each observation period, this association does not account for the fact that the post-initiation observation necessarily occurred at older ages than the pre-initiation observation.

In a sensitivity analysis additionally adjusting for corrective osteotomies with telescopic intramedullary rodding procedures, the association between the post-pamidronate initiation period and fracture incidence remained essentially unchanged (IRR 0.49, 95% CI 0.35–0.68; *p* < 0.001). Corrective osteotomies with telescopic intramedullary rodding procedures were not in-dependently associated with fracture incidence (IRR 1.02, 95% CI 0.67–1.54; *p* = 0.936). Because the timing of orthopedic interventions relative to individual fractures could not be modeled as a time-dependent exposure, this analysis should be interpreted as exploratory.

## 4 Discussion

### 4.1 Principal findings

The present multicenter retrospective cohort study, conducted across three tertiary pediatric referral centers in Bucharest over a 20-year study period (during the 2001–2021 study period), provides real-world data on fracture incidence before and after initiation of intravenous pamidronate therapy in Romanian children with OI. Across the cohort, 198 fractures were documented during 250.3 person-years before pamidronate initiation and 208 fractures during 359.3 person-years after initiation, corresponding to crude incidence rates of 0.79 and 0.58 fractures per person-year, respectively.

Pamidronate therapy was initiated at a median age of 45 months (IQR, 15.5–95), compared with a median age at OI diagnosis of 24.5 months (IQR, 0.25–81.25). The interval between OI diagnosis and pamidronate initiation observed in our cohort likely reflects the individualized approach to treatment during the study period. Diagnosis of OI did not automatically lead to immediate bisphosphonate therapy. Treatment was initiated according to the subsequent clinical course, the severity of skeletal involvement, treatment availability in the hospital and the clinical judgment of the treating physician. The long study period should also be considered, as access to bisphosphonate therapy and treatment practices evolved over time. Because the specific reason for treatment initiation and its timing could not be retrospectively established for every patient, the contribution of each of these factors cannot be quantified.

The repeated pre- and post-treatment observations contributed by each patient were accounted for using a negative binomial GEE model. After adjustment for Sillence type, sex, and age at pamidronate initiation, fracture incidence remained significantly lower during the post-pamidronate initiation period than during the pre-pamidronate period (IRR 0.49, 95% CI 0.35–0.68; *p* < 0.001). Thus, the post-initiation period was associated with an approximately 51% lower fracture incidence rate.

Fracture incidence also differed according to OI phenotype. Compared with Sillence type I, type III was associated with an approximately two-fold higher fracture incidence (IRR 2.09, 95% CI 1.49–2.92; *p* < 0.001), while type IV was also associated with a higher incidence (IRR 1.72, 95% CI 1.01–2.90; *p* = 0.048). Although the point estimate for type V suggested a higher fracture incidence, the confidence interval was wide and the association was not statistically significant, reflecting the very small number of patients with this rare subtype.

Older age at pamidronate initiation was associated with lower fracture incidence in the multivariable model. However, this finding should not be interpreted as evidence supporting delayed treatment. Age at initiation may reflect several underlying characteristics, including disease severity, OI phenotype, age at diagnosis, referral patterns, and clinical decisions regarding initiation of bisphosphonate therapy.

Overall, fracture incidence was substantially lower after pamidronate initiation in this real-world cohort of children with OI. This association remained significant after accounting for repeated observations within the same patients and relevant clinical characteristics. Although the observational design does not allow us to determine how much of this reduction can be attributed specifically to pamidronate, the magnitude of the difference suggests that the lower fracture burden observed after treatment initiation is clinically relevant.

### 4.2 Comparison with previous literature

The lower fracture incidence observed during the post-pamidronate initiation period is consistent with previous observational studies reporting decreased fracture frequency following intravenous bisphosphonate therapy in children with OI. The landmark study by Glorieux et al. (1998), which first established the efficacy of cyclical intravenous pamidronate in severe pediatric OI, reporting marked improvements in bone mineral density, vertebral morphology, and fracture incidence (19). Subsequent observational studies from Europe and North America have similarly reported reductions in fracture frequency together with improvements in mobility and quality of life following long-term bisphosphonate treatment (21, 35–37).

Nevertheless, the effect of bisphosphonates on fracture prevention remains less consistent than their well-established effect on bone mineral density. A recent Cochrane systematic review concluded that although bisphosphonates consistently increase bone mineral density in pediatric OI, evidence supporting fracture reduction remains limited because of heterogeneous study designs, relatively small sample sizes, and variable treatment protocols (17). More recently, Datir et al. (2025) summarized the available evidence and highlighted that, despite widespread clinical use, high-quality randomized evidence demonstrating a substantial reduction in fracture incidence remains limited (18).

Our findings should therefore be interpreted within this context. Rather than demonstrating definitive treatment efficacy, this study reflects fracture outcomes observed during routine clinical practice over nearly two decades of follow-up in three tertiary referral centers.

Other intravenous bisphosphonates, particularly zoledronic acid, have also been investigated in pediatric OI (26–28, 38). Barros et al. (2012) compared 23 children with OI to pamidronate or zoledronic acid for one year and they reported a that lumbar spine areal BMD increased by 52% from baseline in the pamidronate group and by 75% in the zoledronic acid group, although the difference between treatment groups was not statistically significant. In their study fracture rates also decreased significantly from baseline in both groups (*p* = 0.025 and *p* = 0.048, respectively), but numerical fracture rates and a direct between-group comparison were not reported (26). Vuorimies et al. (2011) evaluated zoledronic acid in 17 children with type I OI treated for 1.0–3.2 years. Median lumbar spine BMD Z-score increased from −2.0 to −0.7 after two years, while 6 patients sustained a total of 10 new long-bone fractures during treatment, compared with 73 fractures documented before treatment in the cohort (27). A more recent meta-analysis that included 9 randomized trials and 595 children found that pamidronate produced the greatest improvement in lumbar spine areal BMD at two years (208.73 mg/cm^2^, 95% CI 60.48–356.98), whereas zoledronic acid showed the largest improvement in lumbar spine Z-score at both one year (1.63 points, 95% CI 0.07–3.19) and two years (1.37 points, 95% CI 0.95–1.79). Evidence regarding fracture prevention remained limited, and no clear superiority of pamidronate or zoledronic acid for fracture outcomes could be established. Their findings highlight differences among bisphosphonate regimens while supporting cautious interpretation of comparisons based primarily on densitometric outcomes (38).

### 4.3 Interpretation of subgroup analyses

In our analysis, type III remained associated with higher fracture incidence after adjustment for treatment period, sex, and age at pamidronate initiation. Type IV was also associated with higher fracture incidence, although the confidence interval approached unity. The association between OI phenotype and fracture incidence observed in our cohort is consistent with the established clinical heterogeneity of OI. Type III is generally characterized by greater skeletal fragility and a higher lifetime fracture burden than type I (39). Yazan et al. (2023) similarly reported variable treatment responses across OI subtypes in a cohort of 65 patients, with more pronounced effects in severe phenotypes (21). The associations observed for type V did not reach statistical significance, but this result should be interpreted cautiously because of the relatively small number of patients in this subgroup (*N* = 2)**.**

The apparent association between older age at pamidronate initiation and lower fracture incidence also requires careful interpretation. At first sight, this finding may appear counterintuitive, as earlier treatment is generally considered desirable in children with severe OI. Several mechanisms may contribute to this observation. Children with milder phenotypes, particularly type I OI, may be diagnosed and referred for treatment later, whereas children with more severe disease may be diagnosed earlier and commence treatment at a younger age (40). Consequently, age at treatment initiation may act partly as a marker of phenotype, diagnostic pathway, and clinical decision-making rather than representing an independent biological determinant of fracture risk. Although adjustment for Sillence type reduced but did not eliminate this association, residual confounding remains likely.

Furthermore, adjustment for age at pamidronate initiation does not fully account for chronological age during the pre- and post-treatment initiation observation periods. By definition, all patients were older during post-treatment initiation follow-up, and fracture risk in pediatric OI may vary with age, growth, mobility, and skeletal maturation (15, 41). Therefore, part of the observed period effect may reflect the natural history of fracture risk rather than pamidronate exposure itself. This remains an important limitation of the present retrospective design.

### 4.4 Potential influence of orthopedic treatment

Current understanding of OI management describes that bisphosphonate therapy, orthopedic surgery, and physiotherapy each contribute independently to functional outcomes (42, 43). In the present cohort, corrective osteotomies with telescopic intramedullary rodding procedures occurred after initiation of pamidronate therapy and therefore represent a potentially relevant time-dependent exposure during the post-initiation period.

In a sensitivity analysis additionally adjusting for whether patients underwent a corrective osteotomies with telescopic intramedullary rodding procedures, the association between period and fracture incidence remained essentially unchanged (IRR 0.49, 95% CI 0.35–0.68; *p* < 0.001). Corrective osteotomies with telescopic intramedullary rodding procedures themselves were not independently associated with fracture incidence (IRR 1.02, 95% CI 0.67–1.54; *p* = 0.936).

However, these findings should not be interpreted as demonstrating an absence of an effect of orthopedic surgery. The available variable captured only whether a patient underwent a Corrective osteotomies with telescopic intramedullary rodding procedures and did not incorporate the exact timing of surgery, number of procedures, anatomical sites involved, or person-time before and after individual interventions. Consequently, orthopedic surgery could not be modeled as a true time-varying exposure, and residual confounding by surgical intervention remains possible.

### 4.5 Clinical implications

Although intravenous pamidronate has been incorporated into international management recommendations for pediatric OI, real-world data from Eastern Europe remain scarce. Romania currently lacks a national OI registry, and published longitudinal multicenter cohorts evaluating long-term treatment outcomes are extremely limited.

The present study therefore contributes valuable regional evidence describing long-term management of pediatric OI in routine clinical practice. The inclusion of patients treated over a 20-year period across three tertiary referral centers reflects real-world treatment patterns and multidisciplinary care, including orthopedic surgery and pharmacological treatment. The observed difference in fracture incidence between the periods before and after pamidronate initiation is clinically relevant, particularly because the analysis incorporated actual fracture counts, person-time at risk, and within-patient correlation. However, the results should complement rather than replace evidence from controlled studies. In particular, the present findings should not be used to infer that the approximately 51% adjusted difference represents the causal magnitude of the effect of pamidronate.

These findings also highlight the importance of integrating pharmacological therapy with orthopedic management, rehabilitation, and longitudinal assessment of skeletal health in children with OI.

### 4.6 Strengths and limitations

To our knowledge, this study represents the largest multicenter Romanian cohort evaluating long-term fracture outcomes following intravenous pamidronate therapy in children with OI. The prolonged observation period, inclusion of patients from three tertiary pediatric referral centers, and evaluation of fracture incidence using absolute fracture counts and person-years of observation provide clinically meaningful real-world data. The before-and-after design reduces some of the variability between patients, as each patient contributed observations to both periods. In addition, the use of a negative binomial GEE model allowed us to account for overdispersion in fracture counts as well as for the correlation between the pre- and post-pamidronate initiation observations from the same patient.

Several limitations of the present study must be acknowledged. The retrospective design is subject to selection bias, information bias, and incomplete data capture. The exclusion of 37 patients from the initial registry of 86 due to incomplete records may have introduced systematic differences between included and excluded patients, potentially limiting the generalizability of our findings.

The absence of a concurrent untreated control group limits causal interpretation of the observed difference between the pre- and post-pamidronate initiation periods. All patients were necessarily older during the post-treatment initiation period, and fracture risk in children with OI may change with age, skeletal maturation, mobility, and the natural history of the disease. Although age at pamidronate initiation was included in the multivariable model, this does not fully account for changes in chronological age during follow-up. Therefore, age-related changes in fracture risk may have contributed to the lower fracture incidence observed after treatment initiation, and the observed association cannot be attributed exclusively to pamidronate therapy.

Another consideration is that inclusion required a documented history of fractures before pamidronate initiation. Patients may therefore have entered treatment following a period of relatively high fracture activity, and regression to the mean may explain part of the subsequent decrease in fracture incidence. Pretreatment person-time was calculated from birth to pamidronate initiation, and fractures occurring before formal diagnosis were included when documented in the available medical history. As a result, ascertainment of fractures during the pretreatment period relied partly on retrospective medical history rather than continuous observation within the participating centers, and fractures managed elsewhere may not have been completely captured. The approximately 51% lower fracture incidence estimated by the adjusted GEE model should therefore be interpreted as an association between the post-pamidronate initiation period and fracture incidence and not as an estimate of the causal effect of pamidronate.

Orthopedic treatment represents another potential source of confounding. Corrective osteotomies with telescopic intramedullary rodding procedures were included in an additional sensitivity analysis and did not materially change the association between treatment period and fracture incidence. However, the available data did not allow the timing, number, anatomical site, and indication of individual procedures to be incorporated as time-dependent variables. Similarly, although physiotherapy was documented, its timing and intensity in relation to pamidronate initiation could not be reliably reconstructed and it was therefore not included in the multivariable model.

Bone mineral density (BMD) data were not systematically available for all patients and were therefore not included in the primary analysis. BMD assessment via dual-energy x-ray absorptiometry (DXA) represents an important surrogate endpoint in bisphosphonate trials and its absence limits our ability to correlate structural bone changes with fracture outcomes (44).

Genetic testing was not performed in the majority of patients (65.3%), reflecting the limited availability of molecular diagnostics in the Romanian healthcare system during the study period. Furthermore, two patients with a clinical diagnosis of OI had negative targeted *COL1A1/COL1A2* testing but did not undergo broader multigene panel or whole-exome sequencing.

### 4.7 Future perspective

Future studies should also evaluate emerging therapies, including sclerostin inhibitors, TGF-*β* inhibitors, and gene-based approaches, which may complement or eventually replace antiresorptive treatment in selected patients (45).

Prospective multicenter registries with standardized data collection protocols are needed to overcome the limitations inherent to retrospective cohort designs, particularly with respect to dosing documentation, BMD monitoring, and radiological fracture ascertainment. The establishment of a national Romanian OI registry would facilitate longitudinal tracking of treatment outcomes, surgical interventions, and functional status, enabling more robust comparative analyses and integration with existing European rare bone disease networks, such as the ERN BOND (European Reference Network for Rare Bone Disorders) (46).

The integration of systematic genetic testing into OI diagnostic pathways is a priority, as molecular classification is increasingly recognized as essential for prognosis, counseling, and therapeutic decision-making (47, 48).

## 5 Conclusions

In this retrospective multicenter cohort of 49 children with osteogenesis imperfecta, fracture incidence was lower during the period following initiation of intravenous pamidronate than during the preceding period. After accounting for person-time, with-in-patient correlation, OI phenotype, sex, and age at treatment initiation using a negative binomial GEE model, the post-pamidronate initiation period remained associated with lower fractures incidence (IRR 0.49, 95% CI 0.35–0.68; *p* < 0.001). Higher fracture incidence was observed in patients with more severe OI phenotypes, particularly type III.

Given the retrospective before-and-after design, the absence of an untreated control group, age-related changes in fracture risk, potential regression to the mean, and incompletely characterized time-dependent orthopedic interventions, the observed association should not be interpreted as establishing a causal effect of pamidronate. These findings provide long-term real-world data from an Eastern European pediatric OI cohort and support prospective longitudinal studies incorporating age-dependent fracture risk, standardized fracture ascertainment, and time-varying treatment and orthopedic exposures.

## Data Availability

The raw data supporting the conclusions of this article will be made available by the authors, without undue reservation.
